# Metastatic Colorectal Cancer Patient With Microsatellite Stability and BRAF^V600E^ Mutation Showed a Complete Metabolic Response to PD-1 Blockade and Bevacizumab: A Case Report

**DOI:** 10.3389/fonc.2021.652394

**Published:** 2021-04-27

**Authors:** Chongkai Fang, Jietao Lin, Tao Zhang, Jiajun Luo, Duorui Nie, Meng Li, Xue Hu, Yating Zheng, Xuewu Huang, Zhiwei Xiao

**Affiliations:** ^1^ First Clinical Medical College, Guangzhou University of Chinese Medicine, Guangzhou, China; ^2^ Lingnan Medical Research Center, Guangzhou University of Chinese Medicine, Guangzhou, China; ^3^ Cancer Center, First Affiliated Hospital of Guangzhou University of Chinese Medicine, Guangzhou, China; ^4^ Oncology Department, Shenzhen Hospital Affiliated to Guangzhou University of Chinese Medicine, Shenzhen, China; ^5^ Medical Department, 3D Medicines Inc., Shanghai, China

**Keywords:** PD-1 inhibitor, anti-angiogenesis, BRAF mutation, colon cancer, microsatellite stability

## Abstract

A vast majority of colorectal cancer (CRC) patients with microsatellite stability (MSS) or proficient mismatch repair (pMMR) are refractory to immunotherapeutic strategies. The current research focusses on the combined treatment strategies for identification and optimization in order to improve the efficacy of immunotherapy among patients with microsatellite stability (MSS), who account for the majority of metastatic colorectal cancer (mCRC) cases. mCRC patients harboring MSS and the *BRAF^V600E^ mutation* show a worse prognosis and barely benefit from immunotherapy. In this report, we discuss the case of a mCRC patient with MSS and *BRAF^V600E^ mutation*, who exhibited significant response to the combined treatment with nivolumab and bevacizumab, and has been exhibiting a progression-free survival (PFS) of more than 17 months. Our findings indicate that combined anti-angiogenic therapy can improve the efficacy of immunotherapy, which results in the prolong survival of the patient. This is a case report on MSS and *BRAF^V600E^* colorectal cancer which presents with a response to immunotherapy and anti-angiogenic therapy.

## Introduction

Colorectal cancer (CRC) is the third most commonly diagnosed and fourth most deadly cancer in the world (1). The US Food and Drug Administration (FDA) has approved immunotherapy for the treatment of chemo-resistant metastatic colorectal cancers (mCRC) in patients with microsatellite instability (MSI) or deficient mismatch repair (dMMR), which account for only 4–5% of mCRC patients (2, 3). In contrast, a vast majority of CRC patients with microsatellite stability (MSS) or proficient mismatch (pMMR) are reported to harbor immunologically “cold” tumors that are refractory to immunotherapeutic strategies (4–6).

BRAF mutation is a common molecular phenotype of mCRCs that correlates with poor prognosis. A meta-analysis has reported that *BRAF^V600E^* mutation leads to the methylation of the MLH1 promoter, thus causing MMR deficiency or leading to MSI, which accounts for 54.6% of the total BRAF - CRC and is particularly common in the elderly population (7). In the CheckMate-142 and Keynote-164 studies, patients with MSI-high (MSI-H) exhibited significant responses to immunotherapy (8, 9). However, limited evidence regarding the benefits of immunotherapy on patients with MSS and BRAF mutations exists. Anti-angiogenic inhibitors can normalize the tumor blood vessels and transform the immunosuppressive state of the tumor microenvironment into an immune-supportive state, thereby, inducing tumor sensitivity to immunotherapy and enhancing the anti-tumor effect of immune checkpoint inhibitor (ICIs) (10, 11). Phase Ib study REGONIVO shows that regorafenib combined with immunotherapy may benefit MSS-CRC patients, possibly because the Vascular Endothelial Growth Factor Receptor (VEGFR) is one of the multiple targets of regorafenib (12). However, there is absence of any single anti-angiogenic targeted drug study that supports this view.

Here, we describe a mCRC patient with MSS and *BRAF^V600E^* mutation who has displayed a remarkable response towards the application of combined immunotherapy and bevacizumab treatment and had a progression-free survival (PFS) of more than 17 months. This case report indicates the potentiality of anti-angiogenic treatment in improving the efficacy of immunotherapy in MSS CRC patients.

## Case Description

**Figure 1:**
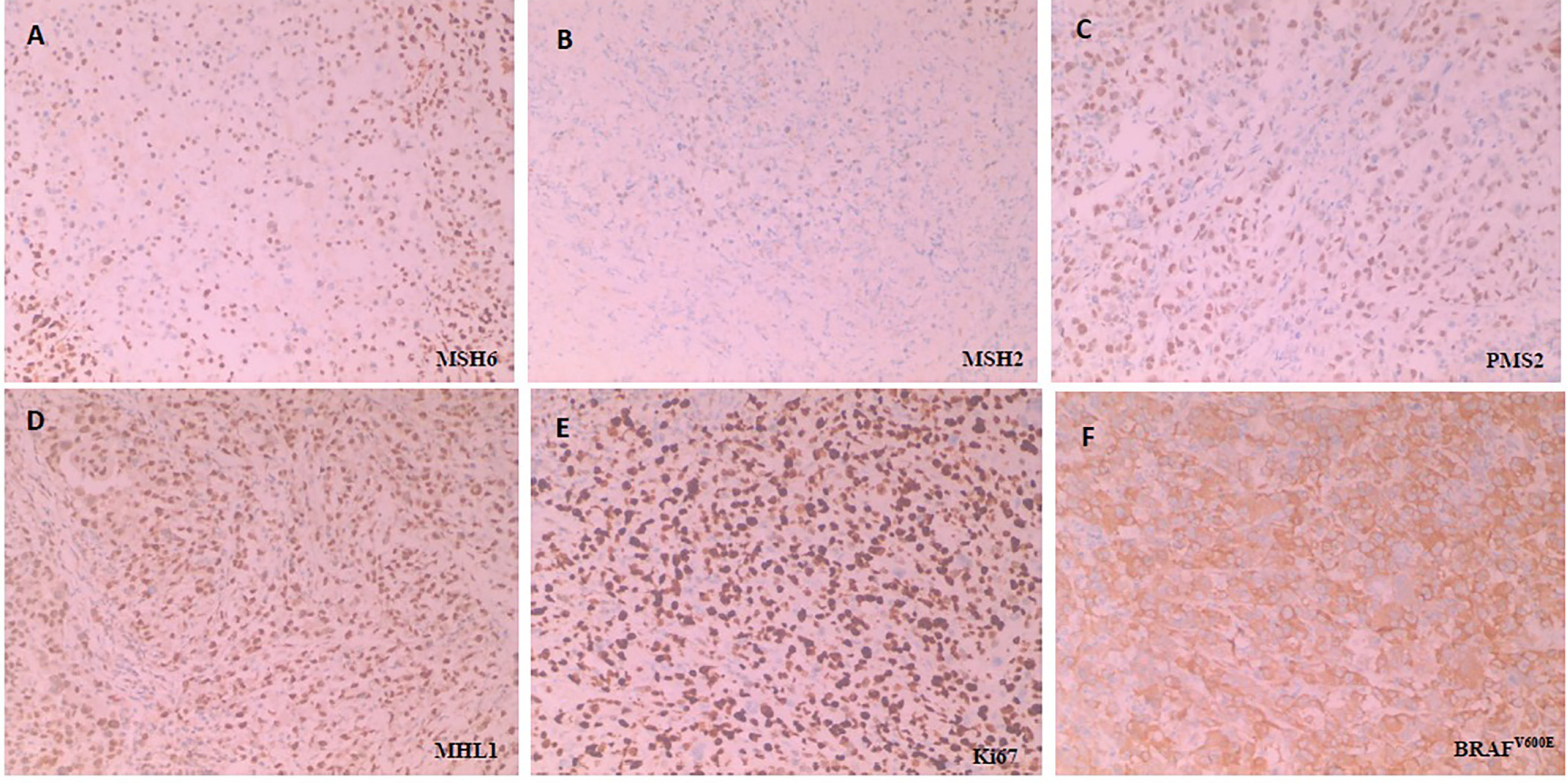
Immunohistochemical staining for detecting MMR, Ki67 and BRAF^V600E^. All the MMR proteins (MSH6, MSH2, PMS2 and MHL1) is no deletion, namely pMMR. BRAF^V600E^ is positive (Anti-BRAF-V600E immunostaining was considered positive if cytoplasmic staining of tumor cells was observed) and Ki67 is proliferative index of 80% (Three or more high-power (40X) fields were selected to calculate the positive staining rate of Ki67 in tumor cells within these fields, and the average value was taken). H&E sample of MMR proteins, including **(A)** MSH6(+), **(B)** MSH2(+), **(C)** PMS2(+) and **(D)** MHL1(+), **(E)** Ki67 and **(F)** BRAF^V600E^, respectively under × 100 magnification.

A 54-year-old female was admitted with right upper abdominal pain, and was diagnosed with right-sided colon cancer through electronic colonoscopy. The diagnosis was further confirmed by biopsy in February 2018. Physical examination was normal and computed tomography (CT) revealed a solid mass in the colonic hepatic flexure with extensive mesenteric and retroperitoneal lymph node metastases. The patient had a history of severe hypertension and her family history showed that her father had bladder cancer and had died in 2019. In May 2018, surgical pathology revealed an invasive, poorly differentiated adenocarcinoma where two out of thirteen mesenteric lymph nodes were positive. Immunohistochemistry (IHC) revealed pMMR, *BRAF^V600E^ mutation* and a Ki67 proliferative index of 80% while the overall pathology report indicated a T3 N2b M0 stage IIIC CRC (**Figure 1**).

**Figure 2:**
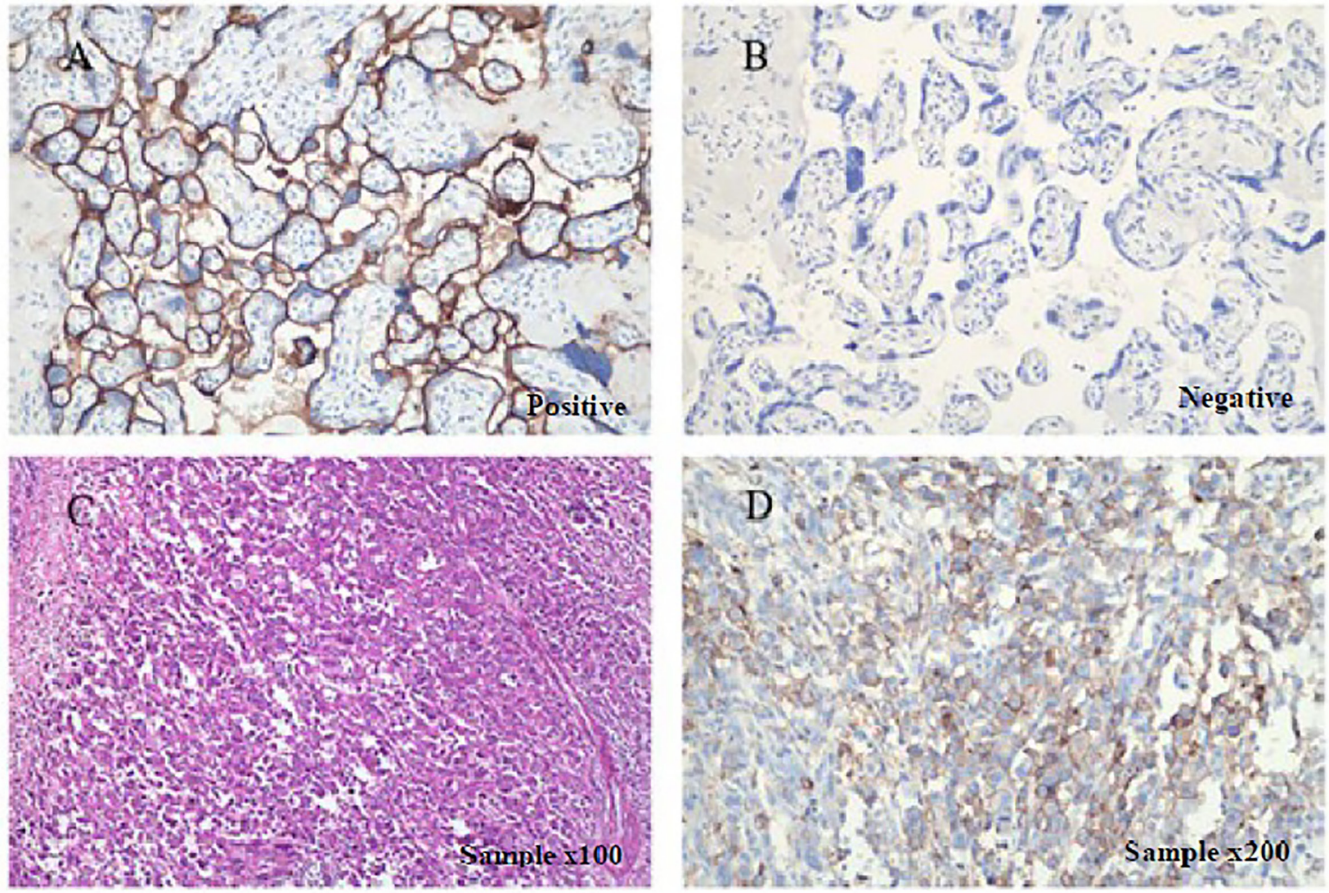
Immunohistochemical staining for detecting PD-L1 expression. PD-L1 is positively expressed in patient’s surgical specimens (tumor proportion score, TPS=30%; combined positive score, CPS=35; PD-L1 expression was determined using Tumor Proportion Score (TPS), the proportion of viable tumor cells showing partial or complete membrane PD-L1 staining at any intensity. PD-L1 expression was also defined using Combined Positive Score (CPS) by dividing the number of PD-L1–stained cells with the total number of viable tumor cells and multiplying by 100). Staining is performed using a Dako 22C3 assay kit following the manufacturer’s instructions. **(A)** Positive and **(B)** negative controls as provided by the kit indicated PD-L1-expressing and non-PD-L1-expressing tissues, respectively under × 200 magnification. **(C)** H&E sample under x100 magnification, and **(D)** IHC sample under × 200.

To seek potential therapeutic options, the patient’s colorectal carcinoma was also subjected to IHC analysis of programmed death-ligand 1 (PD-L1). The tumor proportion score (TPS) reached 30%, whereas the combined positive score (CPS) reached 35 (**Figure 2**). Additionally, upon next generation sequencing (NGS) analysis of the circulating tumor DNA (ctDNA), the patient was identified to MSS (**Supplementary Figure 1**). The final diagnosis was with mCRC with MSS and *BRAF^V600E^* mutation.

**Figure 3:**
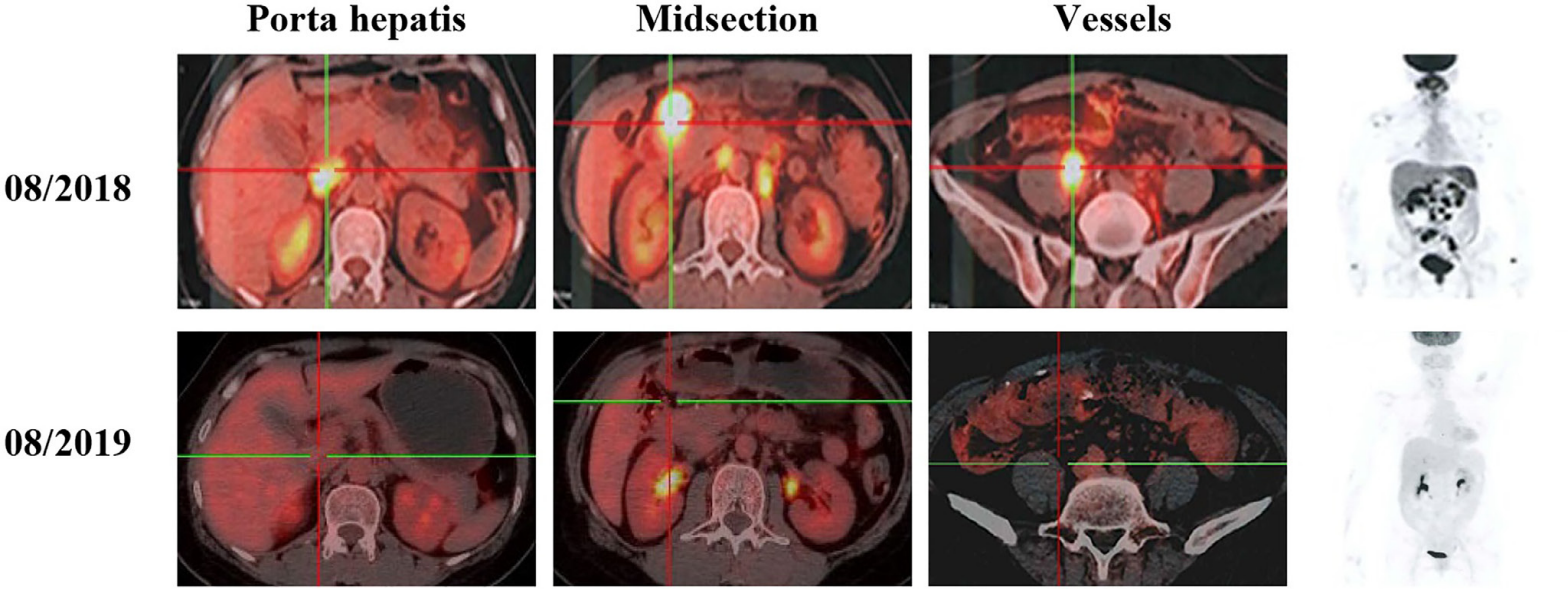
PET-CT scans show the patient’s therapeutic response following a series of treatments, including second-line and third–line therapeutic regimen based on nivolumab.

**Figure 4:**
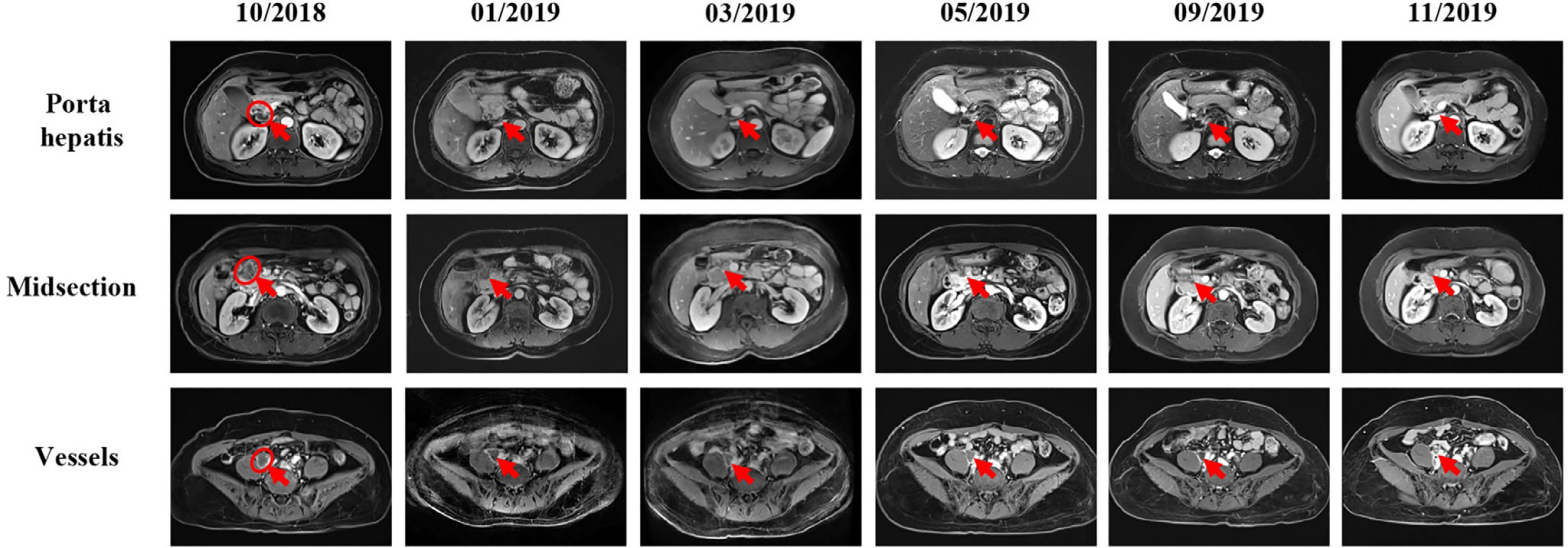
MRI scans show the chronological response of the patient following a series of treatment. The circle represents the area of the target lesion, and arrows indicate the location of the lesions (lymph nodes of porta hepatis, midsection, and vessels). After treatment with FORFIRI and bevacizumab combined, enlarged tumor was observed in October 2018 (lymph nodes of porta hepatis: 23×11mm, midsection: 24×20mm, vessels: 24×18mm). From the change of immunotherapy to November 2019, MRI showed that the tumor was gradually shrinking (The size of hilar hepatis and epigastric mass is not obvious, and the size of vessel is about 16×15mm.).

The patient initially received four cycles of neoadjuvant chemotherapy (mFOLFOX6: oxaliplatin, 150 mg; d1 + 5-fluorouracil, 0.5 g; d1 + maintenance dose of 3.25 g for 46 h). Subsequently, she underwent a right hemicolectomy procedure on May 8, 2018. She received four cycles of mFOLFOX6 chemotherapy followed by her surgery. However, positron emission tomography - computed tomography (PET-CT) showed the disease progression as developed retroperitoneal lymph node metastases (29 × 23 mm) (**Figure 3**). According to the result of PET-CT, the patient was switched on to FOLFIRI chemotherapy in combination with bevacizumab for four cycles. After two months, the magnetic resonance imaging (MRI) scans showed that the disease progressed due to the enlargement (24 × 17 mm) of the lymph node (**Figure 4**). Due to the poor condition of the patient, she agreed to adopt the treatment option of programmed cell death protein 1 (PD-1) inhibitor combined with anti-angiogenic therapy. Consequently, the patient was then prescribed a dosage regimen of nivolumab (140 mg) in combination with bevacizumab (300 mg) every two weeks for 11 cycles (November 23, 2018 to May 5, 2019). Due to the partial response (PR) observed in the MRI scan, the tumor collection diameter (19×16 mm) was reduced by 30.1% (**Figure 4**), subsequently the maintenance therapy was implemented with capecitabine, nivolumab, and bevacizumab. The benefits of this maintenance therapy were confirmed in August 2019 *via* the PET-CT which had recorded the complete metabolic response (**Figure 3**). Considering the observed cutaneous toxicity of grade 2, hypothyroidism and venous thrombosis in the lower extremities, bevacizumab was discontinued in August 2019 and replaced with capecitabine. The entire treatment process has been shown in **Supplementary Figure 2**. As of March 2020, the patient had reached a PFS of more than 17 months since the initiation of the combined nivolumab/bevacizumab therapy. As the patient reported no adverse events during the treatment, so she is still administered nivolumab plus capecitabine.

## Discussion

In this case report, an MSS and BRAF^V600E^ mCRC patient responded well to immunotherapy and bevacizumab, achieving a PFS exceeding 17 months, which allows us to reconsider the therapeutic effect of immunotherapy on such patients.

Tumor with MSS is usually associated with higher malignancy and worse prognosis compared with tumor with MSI-H. For instance, Samowitz suggested that patients with MSS and BRAF mutations have the worst prognosis (13). Additionally, these type of the patients are not sensitive to immunotherapy, thus, only a few treatment options can be considered. It is known that ICIs are almost ineffective for patients with MSS, and clinical research so far has not identified any combination treatment to reverse immunotherapy resistance. While, in 2019 ASCO (American Society of Clinical Oncology), the REGONIVO phase 1b trial reported that nivolumab combined with regorafenib results in an objective response rate of 29% on MSS colorectal cancer patients (12). Regorafenib is a small molecule multi-target inhibitor whose one of the target is VEGFR, that exerts anti-angiogenic effects and increases the ability of T cells to kill tumor cells (14). Anti-angiogenic therapy enhances immunotherapy efficacy by promoting the normalization of blood vessels and the aggregation and infiltration of immune cells, correcting the hypoxic condition, and improving the immune microenvironment (10, 11, 15). In addition, regorafenib also has a target: CSF-1R, which is related to the function of M2 tumor associated macrophage (TAM) with immunosuppressive effect (16). Therefore, from the mechanism, regorafenib can not only inhibit VEGFR, but also play a dual immunoregulation role by inhibiting CSF-1R, effectively regulate the immunosuppressive microenvironment, and better cooperate with PD-1 inhibitors. Hence, the success of REGONIVO suggests that regulating the immune microenvironment might help in improving the response of refractory cancers towards immunotherapy.

Generally speaking, the VEGF down-regulates adhesion factors that aggregate immune cells on endothelial cells, repair damaged blood vessels, and promote blood vessel growth (17). In addition, VEGF inhibits the infiltration of T cells into the tumors and stimulates the proliferation of suppressive immune cells (18–20). Recent studies have suggested that agents targeting VEGF/VEGFR can repair the function of effector T cells and reduce the proliferation, activation, and recruitment of immunosuppressive cells, such as the regulatory T cells (Tregs), tumor-associated macrophages (TAMs), and mast cells (21). Therefore, targeting VEGF may improve the immune response, as well as enhance the efficacy of immunotherapy. Based on above observations upon VEGF, we hypothesized that anti-angiogenic therapy combined with immunotherapy could create a positive feedback loop to increase anti-cancer effects. Unfortunately, we were unable to determine the synergistic effect of VEGF inhibitors and immunotherapy through the REGONIVO study.

Considering the hypotoxicity, VEGF-targeted bevacizumab has been used as an anti-angiogenic inhibitor like regorafenib in our case (22). The success of this case further supports the possibility that VEGF might be the main mechanism of its action. In fact, there are some other studies such as the apatinib combined SHR-1210 of NSCLC, which provides with the same conclusion (23). Owing to the success in the effective treatment of this case, we will continue to monitor the patient’s subsequent changes until the course of the disease progresses. However, there are some limitations in this case. Notably, as this is a case report, the selection might be biased, investigation with a larger sample size is needed. Moreover, the mechanism of drug action has not been further explored, it is hardly to confirm the influence of other target points. And because the tissue samples of patients before and after treatment are not enough for the detection of immune microenvironment related biomarker, it can’t be confirmed that bevacizumab can achieve synergistic effect with PD-1 inhibitors by regulating the tumor immune microenvironment.

**Supplementary Note**, the patient in our case was PD-L1 positive. Even after the patient tested PD-L1 positive, there was no significant benefit shown from immunotherapy, neither in combination therapy nor in monotherapy. Therefore, PD-L1 cannot be used as an effective biomarker to replace or supplement MSI/MMR in the CRC (24, 25).

In summary, we have presented a rare case of MSS mCRC patient showing significantly positive response to nivolumab, a PD-1 inhibitor, bevacizumab, and an angiogenesis inhibitor. Further, large clinical trials are needed to validate the anti-tumor activity of combined nivolumab/bevacizumab therapy.

## Data Availability Statement

The original contributions presented in the study are included in the article/**Supplementary Material**. Further inquiries can be directed to the corresponding authors.

## Ethics Statement

The studies involving human participants were reviewed and approved by Research Ethics Committee of the First Affiliated Hospital of Guangzhou University of Chinese Medicine (ZYYEC-ERK[2021]016). The patients/participants provided their written informed consent to participate in this study. Written informed consent was obtained from the individual(s) for the publication of any potentially identifiable images or data included in this article.

## Author Contributions

Study conception and design: XwH, CF, and JtL. Data acquisition: ZX, TZ, and JjL. Manuscript writing: CF, XH, and YZ. Literature review: DN and ML. All authors have read and approved the manuscript before submission and agreed to be responsible for all aspects of this work. All authors contributed to the article and approved the submitted version.

## Funding

This study was supported by the Guangzhou Science and Technology Bureau (201904010396) and the National Natural Science Foundation of China (81573780).

## Conflict of Interest

XH and YZ were employed by Medicines Inc.

The remaining authors declare that the research was conducted in the absence of any commercial or financial relationships that could be construed as a potential conflict of interest.

## Abbreviations

ASCO, American Society of Clinical Oncology; CEA, carcinoembryonic antigen; CPS, combined positive score; CRC, colorectal cancer; CT, computed tomography; dMMR, mismatch repair-deficient; FDA, Food and Drug Administration; FOLFIRI, 5-fluorouracil, leucovorin and irinotecan; mFOLFOX6, 5-fluorouracil, leucovorin and oxaliplatin; HLA, human leukocyte antigen; ICD, immunogenic cell death; ICI, immune checkpoint inhibitor; mCRC, colorectal cancer; IHC, Immunohistochemistry; MRI, magnetic resonance imaging; MSI, microsatellite instable; MSS, microsatellite stability; NGS, next-generation sequencing; PD-1, programmed cell death protein 1; PD-L1, programmed death-ligand 1; PET-CT, positron emission computed tomography; PFS, progression free survival; pMMR, mismatch repair-proficient; POLE, polymerase epsilon; PR, partial response; SUV, standardized uptake value; TIL, tumor-infiltrating lymphocyte; TPS, tumor proportion score; TMB, tumor mutational burden; TME, tumor microenvironment; Tregs, recruitment of regulatory T cells; TAMs, tumor-associated macrophages; VEGFR, Vascular Endothelial Growth Factor Receptor.
