# Supplementary material for: Metastatic Colorectal Cancer Patient With Microsatellite Stability and BRAFV600E Mutation Showed a Complete Metabolic Response to PD-1 Blockade and Bevacizumab: A Case Report
Source: Front Oncol. 2021 Apr 27;11:652394. doi: 10.3389/fonc.2021.652394 (PMC8112237; doi:10.3389/fonc.2021.652394)
Supplement: Supplementary file 2 [file DataSheet_2.pdf]

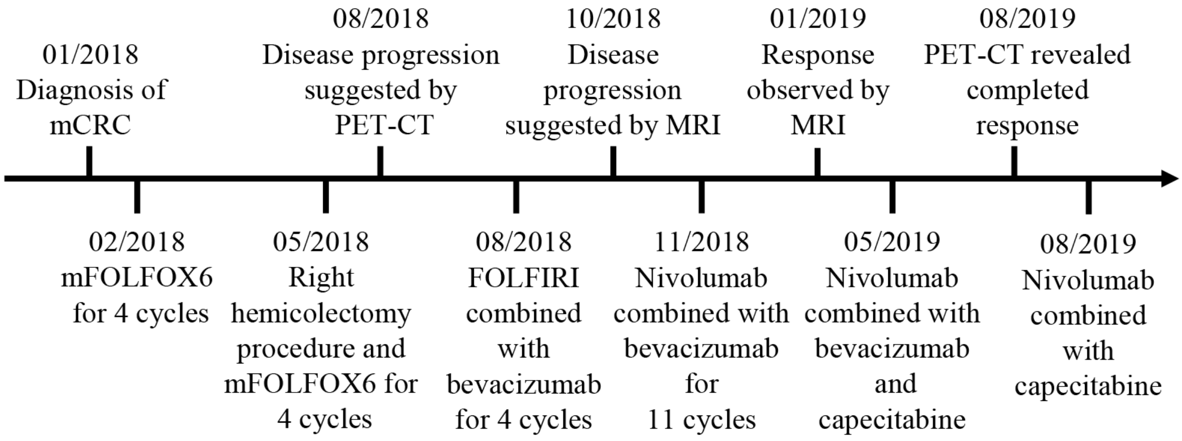


**Supplementary Figure 2.** Schematic diagram showing the time course of patient's diagnosis and treatment.
